# Identification of potentially safe promising fungal cell factories for the production of polyketide natural food colorants using chemotaxonomic rationale

**DOI:** 10.1186/1475-2859-8-24

**Published:** 2009-04-27

**Authors:** Sameer AS Mapari, Anne S Meyer, Ulf Thrane, Jens C Frisvad

**Affiliations:** 1 Center for Microbial Biotechnology, Department of Systems Biology, Building 221, Søltofts Plads, Technical University of Denmark, DK-2800 Kgs. Lyngby, Denmark; 2 Bioprocess Engineering Section, Department of Chemical and Biochemical Engineering, Building 229, Søltofts Plads, Technical University of Denmark, DK-2800 Kgs. Lyngby, Denmark

## Abstract

**Background:**

Colorants derived from natural sources look set to overtake synthetic colorants in market value as manufacturers continue to meet the rising demand for clean label ingredients – particularly in food applications. Many ascomycetous fungi naturally synthesize and secrete pigments and thus provide readily available additional and/or alternative sources of natural colorants that are independent of agro-climatic conditions. With an appropriately selected fungus; using in particular chemotaxonomy as a guide, the fungal natural colorants could be produced in high yields by using the optimized cultivation technology. This approach could secure efficient production of pigments avoiding use of genetic manipulation.

**Results:**

Polyketide pigment producing ascomycetous fungi were evaluated for their potential as production organisms based on *a priori *knowledge on species-specific pigment and potential mycotoxin production and BioSafety level (BSL) classification. Based on taxonomic knowledge, we pre-selected ascomycetous fungi belonging to *Penicillium *subgenus *Biverticillium *that produced yellow, orange or red pigments while deselecting *Penicillium marneffei*; a well known human pathogen in addition to other mycotoxigenic fungi belonging to the same group. We identified 10 strains belonging to 4 species; *viz*. *P. purpurogenum, P. aculeatum, P. funiculosum*, and *P. pinophilum *as potential pigment producers that produced *Monascus*-like pigments but no known mycotoxins. The selection/deselection protocol was illustrated in the pigment extracts of *P. aculeatum *IBT 14259 and *P. crateriforme *IBT 5015 analysed by HPLC-DAD-MS. In addition, extracellular pigment producing ability of some of the potential pigment producers was evaluated in liquid media with a solid support and N-glutarylmonascorubramine was discovered in the partially purified pigment extract of *P. purpurogenum *IBT 11181 and IBT 3645.

**Conclusion:**

The present work brought out that the use of chemotaxonomic tools and *a priori *knowledge of fungal extrolites is a rational approach towards selection of fungal polyketide pigment producers considering the enormous chemical diversity and biodiversity of ascomycetous fungi. This rationale could be very handy for the selection of potentially safe fungal cell factories not only for polyketide pigments but also for the other industrially important polyketides; the molecular and genetic basis for the biosynthesis of which has not yet been examined in detail. In addition, 4 out of the 10 chemotaxonomically selected promising *Penicillium *strains were shown to produce extracellular pigments in the liquid media using a solid support indicating future cell factory possibilities for polyketide natural food colorants.

## Background

Due to the global increase in the manufacture of processed foods and the continued consumer demands for natural food ingredients, the market for natural colorants for food use is estimated to grow [1]. Currently, the vast majority of the natural food colorants permitted in the European Union and the United States are derived by extraction of the pigments from raw materials obtained from the flowering-plants of the kingdom Plantae [2]. The production of many existing natural colorants of plant origin has a disadvantage of dependence on the supply of raw materials, which are influenced by agro-climatic conditions – in addition, their chemical profile may vary from batch-to-batch. Moreover, many of the pigments derived from the contemporary sources are sensitive to heat, light, and oxygen, and some may even change their colour in response to pH changes as in case of anthocyanins [3]. Many ascomycetous fungi naturally synthesize and secrete pigments and may thus provide a more reliable source for natural, "organic" food colorants with improved functionalities [4]. The diversity of fungal pigments is not only found in their chemical structures but also in the colour range of these pigments that may add new or additional hues to the colour palette of the existing colorants derived from contemporary sources [5].

With an appropriately selected fungus the fungal natural colorants; unlike flowering plants, plant cell or algal sources of colorants, could be produced in high yields by using the available cultivation technology without potential genetic manipulation as tougher legislation and sceptical attitude of consumers make it rather difficult for the acceptance of genetically modified food. The controlled cultivation of pigment producing fungi in bioreactors has the potential to compete with any other means of production and can potentially provide unlimited quantities of colourings provided that imperative toxicological studies are carried out. However, a first requirement is that the potential fungus producing the pigment(s) is non-toxigenic under the given broader range of production conditions and is non-pathogenic to humans. The ability of filamentous fungi to co-produce mycotoxins along with industrially useful extrolites, e.g. as in case of citrinin produced by some of the pigment and statin producing *Monascus *species [6], is a major bottleneck in their approval by the legislative authorities. Some of the pigment producers, for instance, *Penicillium marneffei*, could even be human pathogens [7]. Different cultivation media have been shown to induce production of different pigments [5]; a systematic evaluation of the effect of different media components on pigment production – and/or a better understanding of the factors inducing pigment production in fungi – is currently highly warranted in order to optimize the pigment production.

Another challenge in the fungal production of pigments to be used as natural colorants is whether the pigment producer is able to produce pigments in the liquid media or not. Fungal pigments like most other secondary metabolites are preferably produced on solid substrates as these substrates provide support for the fungal mycelia. Therefore, we employed a combination of solid and liquid cultivation technology whereby the fungal mycelia were allowed to grow on a solid support like Lightweight Expanded Clay Aggregates (LECA). The use of LECA has been described as fungal support matrix by Nielsen et al. [8]. Another advantage in this technique was the easier separation of biomass and the fermentation broth with secreted pigments.

Thus, it is of utmost importance to address the question of how to rationally select a promising fungal pigment producer – meeting the above requirements – considering the enormous chemical and biodiversity of fungi. Moreover, the molecular and genetic basis for the polyketide pigment biosynthesis in fungi have not yet been examined in detail leaving genomic approaches for screening unfeasible at this point of time.

In the light of this, we provide a comprehensive list, based on chemotaxonomy, of a majority of the polyketide pigment producing ascomycetous fungi, their pigment composition, and the toxigenic potential with a list of known coloured as well as uncoloured toxic metabolites. Based on taxonomic knowledge, we focussed on pigment producing ascomycetous fungi belonging to *Penicillium *subgenus *Biverticillium *that produced yellow-orange-red pigments while human pathogenic and mycotoxigenic strains belonging to the same group were deselected. We exemplify our chemotaxonomic selection and/or de-selection approach in two Penicillia *viz. Penicillium aculeatum *and *Penicillium crateriforme *grown on 5 different complex solid media to identify potential pigment producers that produced known pigments with or without mycotoxin. The aim of this study was to prove the pertinent use of chemotaxonomic trait of metabolite profiling by powerful tools as HPLC-DAD-MS to come up with promising polyketide pigment producing cell factories that are neither known to be human pathogens nor to produce any known mycotoxins. The ultimate goal is to establish such potentially safe fungal cell factories for the production of polyketide natural colorants. In addition, we also evaluated 4 potential pigment producers for their pigment producing ability in the liquid media using solid support.

## Results

### Chemotaxonomic selection/de-selection for potential polyketide pigment producing ascomycetous fungi

**Figure 1:**
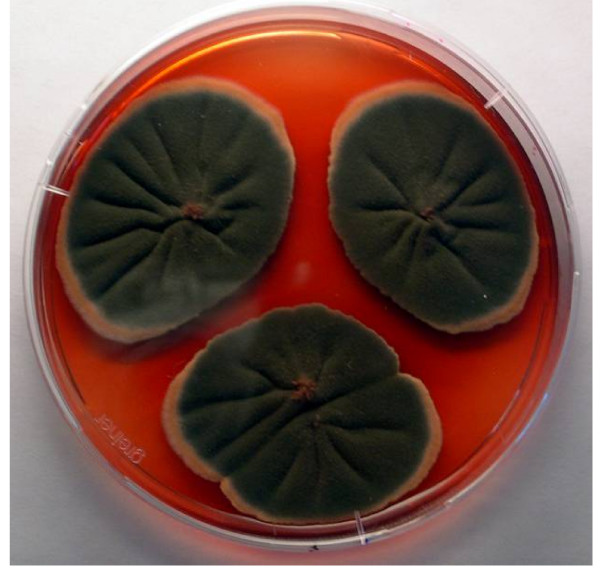
***Penicillium purpurogenum *IBT 11180 on YES agar after 7 days of incubation showing extracellular pigment production**.

**Figure 2:**
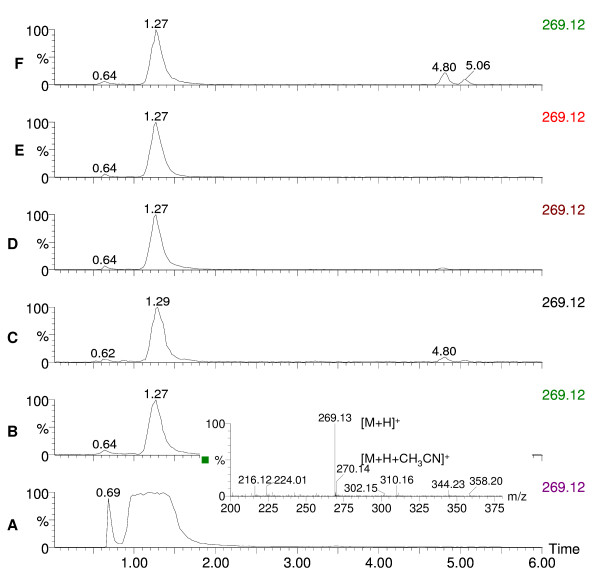
**Chromatograms of rugulovasine A and B and pigment extracts of *Penicillium crateriforme***. The extracted ion chromatograms (m/z 269.12) of standard rugulovasine A and B and pigment extracts of *P.crateriforme *IBT 5015 grown on 5 different solid agar media depict the presence of rugulovasine A and B with its mass spectrum. 2A, standard rugulovasine A and B; 2B, pigment extract from CYA; 2C, pigment extract from MEA; 2D, pigment extract from PD; 2E, pigment extract from OAT; 2F, pigment extract from YES.

**Figure 3:**
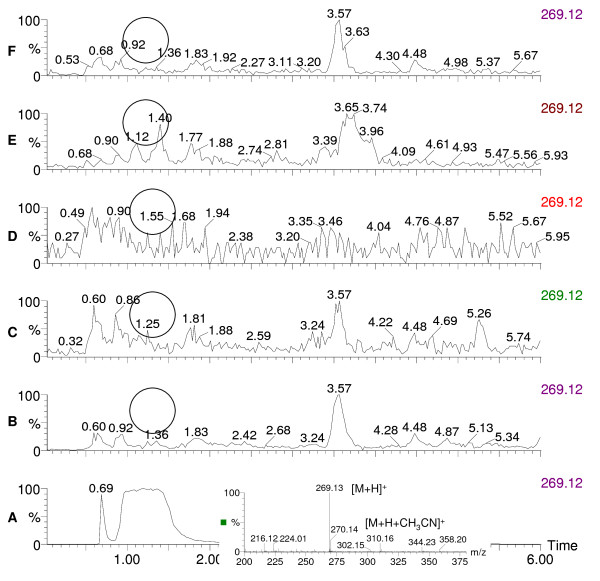
**Chromatograms of rugulovasine A and B and pigment extracts of *Penicillium aculeatum***. The extracted ion chromatograms (m/z 269.12) of standard rugulovasine A and B and pigment extracts of *P. aculeatum *IBT 14259 grown on 5 different solid agar media depict the absence of rugulovasine A and B with its mass spectrum. 3A, standard rugulovasine A and B; 3B, pigment extract from YES; 3C, pigment extract from PD; 3D, pigment extract from OAT; 3E, pigment extract from MEA; 3F, pigment extract from CYA.

**Figure 4:**
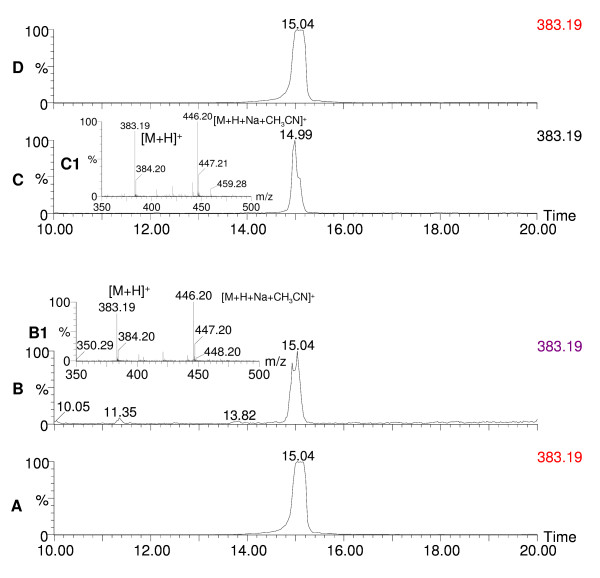
**Chromatograms of monascorubrin and pigment extracts of *Penicillium aculeatum *and *Penicillium crateriforme***. The extracted ion chromatogram (m/z 383.19) of standard monascorubrin and pigment extracts of *P. aculeatum *IBT 14259 and *P. crateriforme *IBT 5015 on CYA depict the presence of monascorubrin with its mass spectrum. Bottom panel A, standard monascorubrin; B, pigment extract of *P. crateriforme *IBT 5015 on CYA; B1, mass spectrum of monascorubrin. Top panel C, pigment extract of *P. aculeatum *IBT 14259 on CYA; C1, mass spectrum of monascorubrin; D, standard monascorubrin.

Many species of *Penicillium *and *Aspergillus *and their teleomorphs have been metabolically profiled for production of pigments and mycotoxins at our research center. The major pigment profiles are listed in Table 1 with citations to relevant literature at the species level [4,5,9-23]. In addition to the well-known pigment producing *Monascus *species used in Asia many species of *Fusarium, Alternaria*, and *Epicoccum *are also polyketide pigment producers, and strains from these genera have also been profiled at our research center. Detailed profiles are listed in Table 2 with relevant citations at the species level [5,24-28]. To illustrate the diversity and the potential of possible pigments producers Table 2 also gives citations to pigment production by *Cladosporium*, *Cordyceps*, *Curvularia*, *Drechslera *and *Paecilomyces *species [4,29-31]. This *a priori *knowledge on potential mycotoxin production and the evaluation of pathogenic potential on the basis of BioSafety Level (BSL) classification [32] formed the basis of our pre-selection/deselection of pigment producers. Strains of the species belonging to *Penicillium *subgenus *Biverticillium *(Table 1) were known to produce copious amounts of yellow, orange and red pigments on solid media; one of the representative red pigment producers is shown in Figure 1. These strains of the species were chosen to be studied. We deselected strains of the 4 species *viz. P. islandicum, P. marneffei, P. variabile *and *P. rugulosum *including the teleomorph *Talaromyces macrosporus *being either pathogenic and/or mycotoxigenic. We screened 11 strains belonging to the rest of the 5 species *viz*. *P. purpurogenum, P. crateriforme, P. aculeatum, P. funiculosum*, and *P. pinophilum *where by *P. crateriforme *served as a positive control in which the presence of mycotoxin was known *a priori *but the pigment was still uncharacterized. The chromatographic analyses of two representative pigment extracts that illustrate our selection/de-selection approach are presented in Figure 2, Figure 3 and Figure 4. Figure 2 depicts the extracted ion chromatogram (m/z 269.12) obtained by positive ESI chromatography using authentic standard of rugulovasine A and B. The pigment extracts of *Penicillium crateriforme *IBT 5015 grown on CYA (Figure 2B), MEA (Figure 2C), PD (Figure 2D), OAT (Figure 2E), and YES (Figure 2F) media were found to be positive for the presence of rugulovasine A and B. Figure 3 depicts the absence of rugulovasine A and B in the pigment extracts of *Penicillium aculeatum *IBT 14259 grown on YES (Figure 3B), PD (Figure 3C), OAT (Figure 3D), MEA (Figure 3E), and CYA (Figure 3F). Both of these Penicillia were found to produce a well-known orange *Monascus *pigment, monascorubrin, in CYA as shown in Figure 4B and Figure 4C by means of the extracted ion chromatogram (m/z 383.19) and mass spectrum, obtained by positive ESI chromatography using authentic standard. Since *Penicillium crateriforme *IBT 5015 produced rugulovasine A and B, in addition to rubratoxin, and spiculisporic acid [9], it was deselected, while *Penicillium aculeatum *IBT 14259 was selected. The rest of the 10 strains (Table 3) were found to produce known *Monascus*-like azaphilone pigments but none of the known mycotoxins such as rubratoxin, luteoskyrin, islanditoxin, rugulosin, cyclochlorotine, erythroskyrin, emodin, spiculisporic acid, and rugulovasine A and B associated with this group of fungi [9]. To the best of our knowledge, the genes or the enzymes involved in the biosynthesis of the above-mentioned mycotoxins are not yet characterized.

**Table 1:** Comprehensive list of polyketide pigment producing *Penicillium *and *Aspergillus *species and their known teleomorphs.

**Fungal species**	**Pigment composition (colour)**. **Toxic colored compounds in bold**	**Major known mycotoxic uncolored metabolites**	**BioSafety Level (BSL) classification^1^**[32]	**Comment^2^**	**Ref**.
***Penicillium *subgenus *Penicillium***					
*P. atramentosum*	Uncharacterized dark brown	Roquefortine C Rugulovasine A & B	unknown	No	[10]
*P. atrosanguineum*	Phoenicin (red) Uncharacterized yellow and red	unknown	unknown	TBI	[11]
*P. atrovenetum*	Atrovenetin (yellow) Norherqueinone (red)	beta-Nitropropionic acid	unknown	No	[12]
*P. aurantiogriseum*	Uncharacterized	Nephrotoxic glycopeptides Penicillic acid Verrucosidin	1	No	[10]
*P. brevicompactum*	Xanthoepocin (yellow)	Botryodiploidin Mycophenolic acid	1	No	[10]
*P. chrysogenum*	Sorbicillins (yellow) Xanthocillins (yellow)	Roquefortine C	1	No	[10]
*P. citrinum*	Anthraquinones (yellow) **Citrinin **(yellow)		1	No	[4]
*P. cyclopium*	**Viomellein **(reddish-brown) **Xanthomegnin **(orange)	Penicillic acid	unknown	No	[10]
*P. discolor*	Uncharacterized	Chaetoglobosin A, B & C	unknown	No	[10]
*P. echinulatum*	Uncharacterized (yellow)	Territrems	unknown	No	[10]
*P. flavigenum*	Xanthocillins	unknown	unknown	TBI	[10]
					
*P. freii*	**Viomellein **(reddish-brown) **Vioxanthin** **Xanthomegnin **(orange)		unknown	No	[10]
*P. herquei*	Atrovenetin (yellow) Herqueinones (red and yellow)	unknown	unknown	Yes	[13]
*P.oxalicum*	Arpink red™- anthraquinone derivative (red) **Secalonic acid D **(yellow)		unknown	No	[3,14]
*P. paneum*	Uncharacterized (red)	Botryodiploidin Patulin Roquefortine C	unknown	No	[10]
*P. persicinum*	Uncharacterized (Cherry red)	Roquefortine C	unknown	No	[10]
*P. viridicatum*	**Viomellein **(reddish-brown) **Vioxanthin** **Xanthomegnin **(orange)	Penicillic acid Viridic acid	unknown	No	[10]
***Talaromyces *(anamorph *Penicillium *subgenus *Biverticillium*)**					
*T. macrosporus*	Mitorubrin (yellow)	Duclauxin Islanditoxin	unknown	No	[15]
*P. aculeatum*	Uncharacterized		unknown	Yes	[un- pub-lished]
*P. crateriforme*	Uncharacterized	Rubratoxin Rugulovasine A &B Spiculisporic acid	unknown	No	[16]
*P. funiculosum*	Uncharacterized		unknown	Yes	[17]
*P. islandicum*	**Emodin **(yellow) **Erythroskyrin **(orange-red) **Luteoskyrin **(yellow) **Skyrin **(orange)	Cyclochlorotine Islanditoxin Rugulosine Rugulovasine A & B	unknown	No	[16]
*P. marneffei*	Mitorubrinol Monascorubramine (purple-red) Purpactin Rubropunctatin (orange) **Secalonic acid D **(yellow)		3	No	[un- published]
*P. pinophilum*	Uncharacterized		unknown	Yes	[17]
*P. purpurogenum*	Mitorubrin (yellow) Mitorubrinol (orange-red) PP-R (purple red) Purpurogenone (yellow-orange)		1	Yes	[5,16,18]
*P. rugulosum*	**Rugulosin **(yellow)		1	No	[9,16]
*P. variabile*	**Rugulosin **(yellow)		unknown	No	[9,16]
***Eurotium *(anamorph *Aspergillus *subgenus *Aspergillus*)**					
*E. amstelodami*	Auroglaucin (orange) Erythroglaucin (red) Flavoglaucin (yellow) **Physcion **(yellow)	Echinulin	1	No	[9]
*E. chevalieri*	Auroglaucin Erythroglaucin Flavoglaucin **Physcion **(yellow)	Echinulin	1	No	[9]
*E. herbariorum*	Aspergin (yellow) Flavoglaucin (yellow) **Physcion **(yellow)	Echinulin	1	No	[9]
***Aspergillus *section *Circumdati***					
*A. ochraceus*	**Viomellein **(reddish-brown) **Vioxanthin** **Xanthomegnin **(orange)	Ochratoxin A Penicillic acid	1	No	[19]
*A. melleus*	**Rubrosulphin **(red) **Viomellein **(reddish-brown) **Viopurpurin **(purple) **Xanthomegnin **(orange)		unknown	No	[19]
*A. sulphureus*	**Rubrosulphin **(red) **Viomellein **(reddish-brown) **Viopurpurin **(purple) **Xanthomegnin **(orange)		unknown	No	[19]
*A. westerdijkiae*	**Rubrosulphin **(red) **Viomellein **(reddish-brown) **Viopurpurin **(purple) **Xanthomegnin **(orange)	Ochratoxin A Penicillic acid	unknown	No	[19]
***Aspergillus *section *Nigri***					
*A. niger*	Flavioline (orange-red), Nnaptho-γ-pyrones (yellow)	Fumonisins Ochratoxin A	1	No	[20]
*A. sclerotioniger*	Uncharacterized yellow	Ochratoxin A	unknown	No	[20]
***Emericella *(anamorph *Aspergillus *subgenus *Nidulantes*, section *Nidulantes *and section *Versicolores*)**					
*Em. falconensis*	Falconensins C-H (yellow) Falconensones (Yellow) Zeorin (yellow)	unknown	unknown	TBI	[21]
*Em. purpurea*	Epurpurins A-C (yellow)	unknown	unknown	TBI	[22]
*A. versicolor*	**Sterigmatocystin **(yellow)		1	No	[23]

^1 ^BSL-1: saprobes or plant pathogens occupying non-vertebrate ecological niches, or commensals. Infections are coincidental, superficial, and non-invasive or mild. BSL-3: pathogens potentially able to cause severe, deep mycoses in otherwise healthy patients.

^2 ^Keys to selection; Yes: preselected as a possible source of pigments, No: not selected as a possible source of pigments, TBI: to be investigated as a possible source of pigments.

The *a priori* species-specific major pigment and/or toxic metabolite profiles and the BioSafety Level (BSL) classification of the fungal species are highlighted and form the basis for selection/de-selection of the species as a potential source of pigment production.

**Table 2:** Selected ascomycetous fungi and their species-specific polyketide pigment and/or toxic metabolite profiles.

**Fungal species**	**Pigment composition (colour)**. **Toxic colored compounds in bold**	**Major known mycotoxic uncolored metabolites**	**BioSafety Level (BSL) classification^1 ^**[32]	**Comment^2^**	**Ref**.
*Fusarium acuminatum*	Antibiotic Y (yellow) Aurofusarin (red)	Enniatins Moniliformin	unknown	No	[24]
*F. avenaceum*	Antibiotic Y (yellow) Aurofusarin (red)	Enniatins Moniliformin 2-amino-14,16-di-methyloctadecan-3-ol	unknown	No	[24]
*F. culmorum*	Aurofusarin (red) Fuscofusarin (yellow) Rubrofusarin (red)	Butenolide Fusarin C Trichothecenes Zearalenone	unknown	No	[24]
*F. fujikuroi*	Bikaverin (red) Norbikaverin (red) O-demethylanhydrofusarubin (red)	Fumonisins Fusaric acid Gibberellins Moniliformin	1	No	[25]
*F. graminearum*	Aurofusarin (red) Rubrofusarin (red)	Butenolide Fusarin C Trichothecenes Zearalenone	unknown	No	[24]
*F. oxysporum*	2,7-dimehoxy-6-(acetoxyethyl)juglone (yellow) Bikaverin (red) Bostrycoidin (red) Nectriafurone (yellow) Norjavanicin (red) O-methyl-6-hydroxynorjavanicin (yellow) O-methylanhydrofusarubin (orange-red) O-methylfusarubin (red) O-methyljavanicin	Fumonisins Fusaric acid Moniliformin	2	No	[25]
*F. poae*	Aurofusarin (red)	Enniatins Fusarin C Trichothecenes	unknown	No	[24]
*F. sambucinum*	Aurofusarin (red)	Trichothecenes	unknown	No	[24]
*F. solani*	Fusarubin (red) Isomarticins (red) O-ethylfusarubin (red) O-methyldihydrofusarubin (red)		2	No	[25]
*F. sporotrichioides*	Aurofusarin (red) Lycopene	Enniatins Trichothecenes	unknown	No	[24]
*F. stilboides*	Antibiotic Y (yellow) Aurofusarin (red) Nectriafurone (yellow)	Enniatins	unknown	No	[un-published]
*F. tricinctum*	Antibiotic Y (yellow) Aurofusarin (red)	Fusarin C Moniliformin	unknown	No	[24]
*F. venenatum*	Aurofusarin (red) Rubrofusarin (red)	Trichothecenes	unknown	No	[24]
*F. verticillioides*	Fusarubin O-demethylfusarubin O-methyljavanicin O-methylsolaniol (orange-red)	Fumonisins Fusaric acid Moniliformin	unknown	No	[25]
*Alternaria dauci*	Uncharacterized (red)	unknown	1	TBI	[26]
*Alt. porri*	Altersolanol A (yellow-orange)	unknown	1	TBI	[26]
*Alt. solani*	Altersolanol A (yellow-orange)	unknown	1	TBI	[26]
*Alt. tomatophila*	Altersolanol A (yellow-orange)	unknown	unknown	TBI	[26]
*Cladosporium cladosporioides*	Calphostins A, B, C, D, I (red)	unknown	1	TBI	[29]
*Cordyceps unilateralis*	3,5 8-TMON* (red) 4-O-methyl erythrostominone (red) Deoxyerythrostominol (red) Deoxyerythrostominone (red) Epierythrostominol (red) Erythrostominone (red)	unknown	unknown	TBI	[30]
*Curvularia lunata*	Chrysophanol (red) Cynodontin (bronze) Helminthosporin (maroon)	unknown	1	TBI	[4]
*Drechslera *spp.	Catenarin (red) Cynodontin (bronze) Helminthosporin (maroon) Tritisporin (redish-brown)	unknown	unknown	TBI	[4]
*Epicoccum nigrum*	Carotenoids Chromanone (yellow) Epicoccarines A & B Epicocconone (fluorescent yellow) Epipyridone (red) Flavipin (brown) Isobenzofuran derivatives (yellow to brown) Orevactaene (yellow)	unknown	unknown	Yes	[5,27,28]
*Paecilomyces sinclairii*	Uncharacterized (red)	unknown	unknown	TBI	[31]
**Polyketide pigment producer of Asia**					
*Monascus pilosus*	**Citrinin **(yellow)		1	Banned in the EU & the US	[6]
*M. purpureus*	Ankaflavin (yellow) **Citrinin **(yellow) Monascin Monascorubramine Monascorubrin Rubropunctamine (purple-red) Rubropunctatin (orange)	Monascopyridine A & B	1	Banned in the EU & the US	[6]
*M. ruber*	Ankaflavin (yellow) **Citrinin **(yellow) Monascin Monascorubramine Monascorubrin Rubropunctamine (purple-red) Rubropunctatin (orange)		1	Banned in the EU & the US	[6]

^1 ^BSL-1: saprobes or plant pathogens occupying non-vertebrate ecological niches, or commensals. Infections are coincidental, superficial, and non-invasive or mild. BSL-2: Species principally occupying non-vertebrate ecological niches, but with a relatively pronounced ability to survive in vertebrate tissue. In severely immuno-compromised patients they may cause deep opportunistic mycoses. Also pathogens causing superficial infections are in this category.

^2 ^Keys to selection; Yes: preselected as a possible source of pigments, No: not selected as a possible source of pigments, TBI: to be investigated as a possible source of pigments.

The *a priori* major metabolite profiles and the BioSafety Level (BSL) classification of the fungal species are highlighted and form the basis for selection/de-selection of the species as a potential source of pigment production.

**Table 3:** Potentially safe and toxin-free cell factories belonging to *Penicillium *subgenus *Biverticillium *that produce *Monascus*-like pigments.

**Fungal species Serial No**.	**IBT Culture Collection #**	**Other culture collection #**	**Source of isolation**	**Pigments identified**	**Known mycotoxins**
*Penicillium purpurogenum*					
**1**.	11180^1,2^	-	Unknown	Monascorubramine^3^, PP-R^1,3^	None
**2**.	11181^2^	CBS 123796	Pepper fruit	N-glutarylrubropunctamine^4^	None
				N-glutarylmonascorubramine^4^	
**3**.	21347	-	Human saliva	PP-R^5^	None
**4**.	23082	RMF 81.01	Soil	PP-R^3^	None
**5**.	3645^2^	IMI 90178	Unknown	N-glutarylmonascorubramine^6^	None
**6**.	3967^2^	NRRL 1147	Unknown	Monascorubramine^7^	None
				Monascin	
*Penicillium aculeatum*					
**7**.	14259	NRRL 2129	Weathering fabric	Monascorubrin^5^	None
**8**.	14263^1^	FRR 1802	Soil	Monascorubrin^1,3^	None
				Xanthomonasin A^1,3^	
				Threonine derivative of rubropunctatin^1,5^	
*Penicillium funiculosum*					
**9**.	3954	NRRL 2119	Unknown	Ankaflavin^8^	None
*Penicillium pinophilum*					
**10**.	13104^1^	ATCC 9644	Radio set	Monascorubrin^1,3^	None

^1^Previously reported [5,33]; ^2 ^Tested for the pigment producing ability in the liquid media and was positive; ^3^On YES Agar; ^4 ^In M1 liquid medium; ^5 ^On CYA agar; ^6 ^In M2 liquid medium; ^7 ^On OAT agar; ^8 ^On MEA agar.

### New potentially safe fungal cell factories for the production of *Monascus*-like pigments

**Figure 5:**
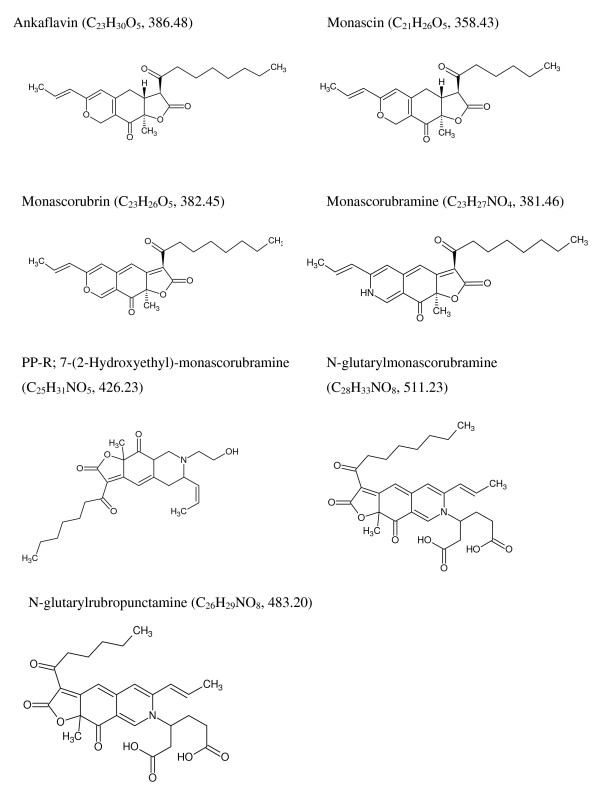
**Structures of the pigments detected and identified in the present study**. Formula and calculated nominal masses are shown in the parentheses.

**Figure 6:**
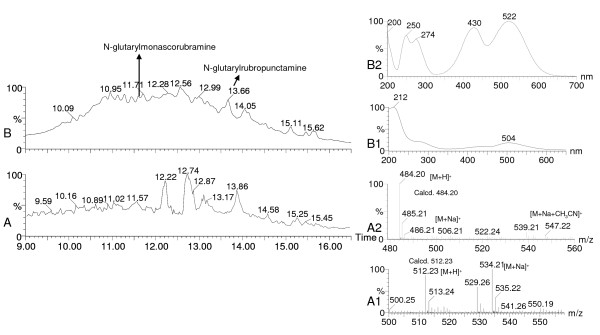
**Presence of N-glutarylmonascorubramine and N-glutarylrubropunctamine in the extracellular pigment extract of *Penicillium purpurogenum *IBT 11181**. The strain was grown in N1 liquid medium with LECA as a solid support. Total ion chromatogram (m/z 100–900), on X-axis time is shown, and on Y-axis % of the ion count (A), and UV-vis chromatogram at 390–700 nm, on X-axis time is shown, and on Y-axis % of the sum of the absorbencies, a relative value (B), mass spectrum (A1) and UV-vis spectrum of N-glutarylmonascorubramine (B1), mass spectrum (A2) and UV-vis spectrum of N-glutarylrubropunctamine (B2).

**Figure 7:**
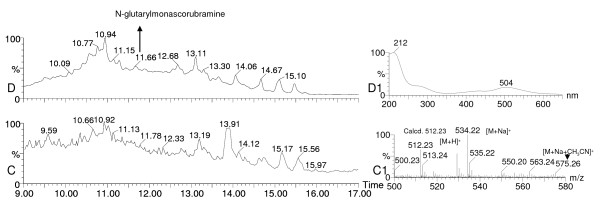
**Presence of N-glutarylmonascorubramine in the extracellular pigment extract of *Penicillium purpurogenum *IBT 3645**. The strain was grown in N2 liquid medium with LECA as a solid support. Total ion chromatogram (m/z 100–900), on X-axis time is shown, and on Y-axis % of the ion count (C), and UV-vis chromatogram at 390–700 nm, on X-axis time is shown, and on Y-axis % of the sum of the absorbencies, a relative value (D), mass spectrum (C1) and UV-vis spectrum of N-glutarylmonascorubramine (D1).

Table 3 brings out 7 strains (2, 3, 4, 5, 6, 7 and 9 in Table 3) as novel producers of *Monascus*-like azaphilone polyketide based pigments such as ankaflavin, monascin, monascorubrin, monascorubramine, PP-R, N-glutarylmonascorubramine, and N-glutarylrubropunctamine (Figure 5), in addition to the 3 strains (1, 8 and 10 in Table 3) reported previously [5,33]. None of these strains were found to produce any known mycotoxins in the mentioned media and under the lab conditions of pigment production. Notably four of the strains tested so far (Table 3), have been found to produce extracellular pigments in the liquid media with LECA as a solid support. For the first time, N-glutarylmonascorubramine and N-glutarylrubropunctamine; the two known water-soluble *Monascus*-pigments were discovered in the extracellular pigment extract obtained from the liquid medium (N1) of *P. purpurogenum *IBT 11181 (Figure 6). The identity was based on the UV-vis and mass spectra (Figure 6). N-glutarylmonascorubramine was also discovered in the extracellular pigment extract of *P. purpurogenum *IBT 3645 obtained from the liquid medium (N2) as shown in Figure 7.

## Discussion

### Chemotaxonomic selection/de-selection for potential polyketide pigment producing ascomycetous fungi

The strains of the species in Table 1 and Table 2 are yet to be investigated as potential production strains on different solid and in liquid media. Some of them may be promising as potentially safe cell factories for pigments with an array of different colors indicating that a lot more fungal biodiversity is yet to be explored for the discovery of novel sources of natural colorants. *Epicoccum nigrum *(Table 2) has been evaluated for pigment production [34], and *Penicillium herquei *(Table 1) has been partially evaluated (data unpublished). They could be potential cell factories for natural green-yellow to yellow colorants. Biosynthetically, a majority of pigments produced by filamentous fungi are polyketide-based (some may involve polyketide-amino acid mixed biosynthesis) and involve complex pathways catalysed by iterative type I polyketide synthases as exemplified in case of pigments produced by *Monascus ruber *[35]. A complete knowledge about the biosynthetic pathway of polyketide pigments including the extensively studied *Monascus *pigments is not yet available. Also, genomic approaches of selection of potential polyketide pigment producers may not be useful at this point of time when none of the fungal polyketide pigment producers are fully genome sequenced yet. Moreover, the problem of annotating correct gene sequences is not to be overlooked especially due to the variation in the domain of polyketide synthases; two or more polyketide synthases are involved in the biosynthesis of such complex secondary metabolites as pigments. Chemotaxonomy uses secondary metabolite profiles of filamentous fungi as the secondary metabolites have a differentiation capability at a genus and species level [36]. This has been used successfully and resulted in a lot of *a priori *knowledge about filamentous fungi – their bio- and chemical diversity, ecological niche, the species-specific metabolite profiles, and optimal media and growth conditions for secondary metabolites (including pigments) production [37]. Phylogeny as a taxonomic tool, whereby the partial sequences of the household genes such as β-tubulin are used, on the other hand, cannot predict the functional trait of the organisms as described by Samson et al. [38]. Therefore, chemotaxonomic selection would form an essential element of high throughput screening programmes as the use of *a priori *knowledge of species-specific metabolite/pigment and/or mycotoxin profiles ensures a quick and efficient way of selecting potentially safe pigment producers from a vast bio- and chemodiversity of filamentous fungi. The purpose of the present work was to demonstrate the chemotaxonomic selection and de-selection approach in the pigment producing fungi whereby specific mycotoxin production was shown in the producer or the non-producer in addition to the *a priori *knowledge of their mycotoxin and pigment profile. Thus, the starting point of our selection approach was the use of chemotaxonomic knowledge about the polyketide pigment producing fungi (Table 1 and Table 2). We used this as a key to show that only a handful of pigment producers are worth exploring as potentially safe fungal cell factories for polyketide pigment production (Table 3). The LC-DAD-MS was successfully used as a powerful tool to check the presence or the absence of mycotoxins using authentic standard in the run and to identify pigments based on UV-vis and mass spectra. Since, pigments have characteristic UV-vis spectra, the use of UV-vis spectra in the identification and de-replication was shown to be quite handy. The two examples (Figure 2, Figure 3 and Figure 4), not only demonstrated our chemotaxonomic selection/de-selection approach but also proved the use of LC-DAD-MS as an efficient chemotaxonomic tool. To the best of our knowledge, this unique and rational approach is not yet demonstrated as a selection criterion for fungal cell factories. Thus, it signifies our findings to emphasize on the appropriate use of chemotaxonomy in the discovery of novel and potentially safe cell factories for the industrially useful polyketides including pigments for food use. However, the toxicological studies are still imperative for the final approval of the product and the process that would entail the use of these pre-selected potentially safe cell factories especially for the food use

### New potentially safe fungal cell factories for the production of *Monascus*-like pigments

Several of the already known *Monascus*-like pigments (Table 3, Figure 4 and Figure 5) were identified in *Penicillium *species that do not produce any known mycotoxins when grown under the laboratory conditions used in this study. It could be noted that these Penicillia are closely related according to Raper and Thom [39], and Pitt [40] and that they are also closely related according to phylogenetic principles [41] but distantly related to *Monascus *species.

The production of extracellular pigments by some of the tested cell factories in the liquid media using a solid support has added to the value of these cell factories as future production strains. N-glutarylmonascorubramine and N-glutarylrubropunctamine have been reported as water-soluble extracellular pigments of *Monascus ruber *[42-44]. The identification of N-glutarylmonascorubramine was based on mass and UV-vis spectra that matched quite well with the previous reports [42,44]. The mass spectra of N-glutarylrubropunctamine matched very well with the one reported by Hajjaj et al. [44]. The UV-vis spectrum of N-glutarylrubropunctamine was very similar to that of N-glutarylmonascorubramine as the two compounds differed only in their aliphatic side chain. Multiple extracellular pigments (Figure 6 and Figure 7) were formed from a complex nitrogen source such as yeast extract (as in case of N1 medium) or a combination of such complex nitrogen sources as corn steep liquor and yeast extract (as in case of N2 medium) and rubropunctatin and/or monascorubrin in the media possibly by Schiff base formation type of reaction mechanism. This mechanism was reported previously [42,44] for the formation of these water soluble pigments from their counter parts which are aminophilic in nature being azaphilones. Hajjaj et al. [44] reported the formation of 4 pigment molecules that constituted 91% of the pigment production when only one nitrogen source (monosodium glutamate) in the medium was used with 20 g/L glucose. Our results indicate formation of much more than 4 pigment components for both of the tested Penicillia (Figure 6 and Figure 7) possibly due to the availability of several amino acids from the complex nitrogen source/s provided in the media. Thus, in the future it is possible to obtain desired number of pigment components by using defined nitrogen sources that would provide specific amino acid/s moiety to be incorporated into the azaphilone pigments to form water soluble extracellular pigments. As these water soluble extracellular pigments are more stable than their orange counterparts [45], this is very significant for the biotechnological production of such natural colorants in these newly discovered cell factories Thus it can be inferred that the discovery of such potentially safe cell factories for polyketide natural food colorants using chemotaxonomic approach is a milestone that would redefine the biotechnological production of food colorants derived from such an agro-independent source of colorants as filamentous fungi. Moreover, this also can tackle the current issue of restricted use of *Monascus *pigments due to the co-production of toxic metabolites.

## Conclusion

In conclusion, we have shown that the use of chemotaxonomic tools and *a priori *knowledge of fungal extrolites is a rational approach towards selection of potentially safe polyketide natural colorant producing fungal cell factories considering the enormous chemical diversity and biodiversity of ascomycetous fungi. This approach could be very handy for the selection of potentially safe fungal cell factories not only for polyketide pigments but also for the other industrially important polyketides; the molecular and genetic basis for the biosynthesis of which has not yet been examined in detail. In addition, 4 out of the 10 chemotaxonomically selected promising *Penicillium *strains were shown to produce extracellular pigments in the liquid media using a solid support; two of the pigments were identified as *Monascus *pigment derivatives N-glutarylrubropunctamine and N-glutarylmonascorubramine, by means of LC-DAD-MS chromatography. Work is underway for the evaluation of a few of these promising cell factories for the controlled and robust production of such polyketide natural food colorants.

## Methods

### Pre-selection of fungi, media, and cultivation conditions

All fungal isolates used in this study were procured from the IBT Culture Collection at Center for Microbial Biotechnology, Technical University of Denmark, Kgs. Lyngby, Denmark. The fungal isolates were listed by the IBT numbers. *Penicillium aculeatum *IBT 14259 and *P. crateriforme *IBT 5015 was cultivated on five different solid media *viz*; Yeast extract sucrose (YES) agar; Malt extract agar (MEA), Oatmeal (OAT) agar, Potato dextrose (PD) agar and Czapek-Dox yeast autolysate (CYA) agar [46]. The cultures were incubated in the dark at 25°C for 7 days.

For the liquid media with the solid support, CZ medium [46] with 0.5% yeast extract (designated as N1) was used in case of *Penicillium purpurogenum *IBT 11181. N2 medium; where the basal medium was kept the same as in CZ medium except that the carbon sources and nitrogen sources were (g/L): potato starch, 2.75; lactose, 5.5; ammonium nitrate, 1.55; corn steep liquor, 1.55, was used for *Penicillium purpurogenum *IBT 3645. The initial pH of the medium was adjusted to 5.5 using 0.1 M HCl. Approximately 8–9 grams of Light Expanded Clay Aggregates (LECA) were used as solid support retained within a tea paper filter (Schur Inventure A/S, Vejle, Denmark, extra fine pores capacity 16.5 g/m^2^, locally purchased) and sterilized by autoclaving. Spores harvested from a week old culture plate (CYA) with a concentration (3 × 10^5^/ml) were inoculated directly onto the LECA contained within the tea filter, which was then transferred into 300 ml baffled Erlenmeyer culture flasks comprising 100 mL of the N1 or N2 media. The experiment was performed in duplicate. The cultures were incubated at 25°C in the dark under shaking conditions (120 rpm).

### Extraction of fungal pigments

In case of solid media, extraction was carried out by a modified version of the micro-scale extraction method [47], where 6 mm plugs were extracted ultrasonically with solvent containing ethyl acetate, dichloromethane, and methanol in a ratio of 3:2:1 (v/v/v) with 1% formic acid. The extract was evaporated to dryness in a rotational vacuum concentrator (RVC; Christ Martin, Osterode, Germany). Residue was re-dissolved in 400 μL methanol, in an ultrasonic bath (Branson 2510, Kell-Strom, Wethersfield, USA) for 10 minutes, and filtered through a 0.45 μm PTFE syringe filter (SRI, Eatontown, New Jersey, USA). This extract was used for chromatographic analysis.

In case of liquid media with solid support, after 7 days of incubation the tea filter comprising the majority of the fungal biomass adhered to LECA was removed from the flask and the fermentation broth containing extracellular pigments was filtered through Whatman filter paper # 1 to remove the residual biomass.

### Evaluation of pigment composition from the fermentation broth

The filtrate was subject to clean-up by solid phase extraction Strata-X-C 33 μm cation mixed mode polymer columns (60 mg 1 mL, Phenomenex, Torrence, California, USA). 1.2 mL of methanol was used for conditioning followed by 1.2 mL of distilled water for calibration. 1.2 mL of samples, acidified with 0.1% phosphoric acid (1: 6 v/v) were loaded in a vacuum manifold, and washed with 0.1% phosphoric acid. Elution of the pigment mixture, bound in the matrix of the cartridges, was carried out using methanol which could elute neutral and acidic components. The pigment extract so obtained was subjected to high resolution LC-DAD-MS analysis.

### Chromatographic analysis

High-resolution LC-DAD-MS was performed on an Agilent HP 1100 liquid chromatograph (LC) system with a photodiode array detector (DAD) and a 50 × 2 mm i.d., 3 μm, Luna C_18 _II column (Phenomenex, Torrance, CA). The LC system was coupled to a LCT orthogonal time-of-flight mass spectrometer (Waters-Micromass, Manchester, U.K.) with a Z-spray electrospray ionization (ESI) source, and a LockSpray probe and controlled by the MassLynx 4.0 software. MS system was operated in the positive ESI mode using a water-acetonitrile gradient system starting from 15% acetonitrile, which was increased linearly to 100% in 20 minutes with a holding time of 5 minutes. The water was buffered with 10 mM ammonium formate and 20 mM formic acid and the acetonitrile with 20 mM formic acid. The instrument was tuned to a resolution > 7000 (at half peak height). The method is well established at our research center for the metabolite profiling of filamentous fungi grown on solid media. It is described by Nielsen et al. [48].

For the extracellular pigments extracted from the liquid media, the solvent system used was water with 0.1% formic acid and acetonitrile with 0.1% formic acid. The gradient started from 5% acetonitrile and increased to 100% in 20 minutes and was hold at 100% for 2 minutes. The MS conditions were the same as mentioned earlier.

### Analysis of LC-DAD-MS Data

The coloured components in the pigment extract were detected in the UV-vis chromatogram of 390–700 nm. The identification of N-glutarylmonascorubramine and N-glutarylrubropunctamine was based on both UV-vis and mass spectra from total ion chromatogram (m/z 100–900) from positive ion electro spray. The UV-vis spectrum was obtained after background subtraction. The DAD-MS data for N-glutarylmonascorubramine and N-glutarylrubropunctamine is shown below:

N-glutarylmonascorubramine was detected as *m/z *512.23 [M + H]^+^and confirmed by the adducts *m/z *534.22 [M + Na]^+ ^and *m/z *575.26 [M + Na + CH_3_CN]^+^. The UV-vis spectrum was λ_max_: 212, with a shoulder at 272, 420 and 504.

N-glutarylrubropunctamine was detected as *m/z *484.20 [M + H]^+^and confirmed by the adducts *m/z *506.21 [M + Na]^+ ^and *m/z *547.22 [M + Na + CH_3_CN]^+^. The UV-vis spectrum was λ_max_: 200, 250, 274, 430 and 522.

## Competing interests

The authors declare that they have no competing interests.

## Authors' contributions

SASM performed the experiments and drafted the manuscript, ASM helped in the writing of the manuscript. UT and JCF contributed to the chemotaxonomic selection/deselection part in Table 1. JCF contributed with his chemotaxonomic expertise in *Penicillium *subgenus *Biverticillium*. All authors read and approved the final manuscript.

## References

[B1] Downham A, Collins P (2000). Colouring our foods in the last and the next millennium. Int J Food Sci Technol.

[B2] Mortensen A (2006). Carotenoids and other pigments as natural colorants. Pure Appl Chem.

[B3] Mapari SAS, Nielsen KF, Larsen TO, Frisvad JC, Meyer AS, Thrane U (2005). Exploring fungal biodiversity for the production of water-soluble pigments as potential natural food colorants. Curr Opin Biotechnol.

[B4] Duran N, Tixeira MFS, de Conti R, Esposito E (2002). Ecological-friendly pigments from fungi. Crit Rev Food Sci Nutr.

[B5] Mapari SAS, Meyer AS, Thrane U (2006). Colorimetric characterization for comparative analysis of fungal pigments and natural food colorants. J Agric Food Chem.

[B6] Wang Y, Ju X, Zhou Y (2005). The variability of citrinin production in *Monascus *type cultures. Food Microbiol.

[B7] Liyan X, Changming L, Xianyi Z, Luxia W, Suisheng X (2004). Fifteen cases of penicillliosis in Guangdong, China. Mycopathologia.

[B8] Nielsen KF, Larsen TO, Frisvad JC (2004). Lightweight expanded clay aggregates (LECA), a new up-scaleable matrix for production of microfungal metabolites. J Antibiot.

[B9] Frisvad JC, Thrane U, Samson RA, Hoekstra ES, Frisvad JC (2004). Mycotoxin production by common filamentous fungi. Introduction to food- and airborne fungi.

[B10] Frisvad JC, Smedsgaard J, Larsen TO, Samson RA (2004). Mycotoxins, drugs and other extrolites produced by species in *Penicillium *subgenus *Penicillium*. Stud Mycol.

[B11] Christensen M, Frisvad JC, Tuthill D (1999). Taxonomy of the *Penicillium miczynskii *group based on morphology and secondary metabolites. Mycol Res.

[B12] Raistrick H, Stössl A (1958). Studies in the biochemistry of microorganisms 104. Metabolites of *Penicillium atrovenetum *G. Smith β-nitropropionic acid, a major metabolite. Biochem J.

[B13] Robinson N, Wood K, Hylands PJ, Gibson TM (1992). Blue pigments of *Penicillium herquei*. J Nat Prod.

[B14] Sardaryan E (2002). Strain of the microorganism *Penicillium oxalicum *var. *Armeniaca *and its application.

[B15] Frisvad JC, Filtenborg O, Samson RA, Stolk AC (1990). Chemotaxonomy of the genus *Talaromyces*. Antonie Van Leeuwenhoek.

[B16] Frisvad JC (1989). The connection between the Penicillia and Aspergilli and mycotoxins with special emphasis on misidentified isolates. Arch Environ Contam.

[B17] van Reenen-Hoekstra ES, Frisvad JC, Samson RA, Stolk AC, Samson RA, Pitt, JI (1990). The *Penicillium funiculosum *complex – well defined species and problematic taxa. Modern concepts in Penicillium and Aspergillus classification.

[B18] Buchi G, White JD, Wogan GN (1965). The structures of mitorubrin and mitorubrinol. J Am Chem Soc.

[B19] Frisvad JC, Frank JM, Houbraken JAMP, Kuijpers AFA, Samson RA (2004). New ochratoxin A producing species of *Aspergillus *section *Circumdati*. Stud Mycol.

[B20] Samson RA, Houbraken JAMP, Kuijpers AFA, Frank JM, Frisvad JC (2004). New ochratoxin A or sclerotium producing species in *Aspergillus *section *Nigri*. Stud Mycol.

[B21] Ogasawara N, Mizuno R, Kawai K (1997). Structures of a new type of yellow pigments, falconenzones A and B, from *Emericella falconensis*. J Chem Soc Perkins Trans.

[B22] Hideyuki T, Koohei N, Ken-ichi K (1996). Isolation and structures of dicyanide derivatives, epurpurins A to C, from *Emericella purpurea*. Chem Pharm Bull (Tokyo).

[B23] Davies JE, Kirkaldy D, Roberts JC (1960). Studies in mycological chemistry. Part VII. Sterigmatocystin, a metabolite of *Aspergillus versicolor *(Vuillemin) Tiraboschi. J Chem Soc.

[B24] Thrane U, Summerell BA, Leslie JF, Backhouse D, Bryden WL, Burgess LW (2001). Developments in the taxonomy of *Fusarium *species based on secondary metabolites. Fusarium Paul E Nelson Memorial Symposium.

[B25] Medentsev AG, Akimenko VK (1998). Naphthoquinone metabolites of the fungi. Phytochem.

[B26] Andersen B, Dongo A, Pryor BM (2008). Secondary metaboliteprofiling of *Alternaria dauci, A. porri, A. solani*, and *A. tomatophila*. Mycol Res.

[B27] Kemami Wangun HV, Hertweck C (2007). Epicoccarines A, B and epipyridone: tetramic acids and pyridine alkaloids from an *Epicoccum *sp. associated with the tree fungus *Pholiota squarrosa*. Org Biomol Chem.

[B28] Lee NH, Gloer JB, Wicklow T (2007). Isolation of chromanone and isobenzofuran derivatives from a fungicolous isolate of *Epicoccum purpurascens*. Bull Korean Chem Soc.

[B29] Kobayashi E, Ando K, Nakano H, Iida T, Ohno H, Morimoto M, Tamaoki T (1989). Calphostins (UCN-1028), novel and specific inhibitors of protein kinase C 1. Fermentation, isolation, physico-chemical properties and biological activities. J Antibiot.

[B30] Unagul P, Wongsa P, Kittakoop P, Intamas S, Srikitikulchai P, Tanticharoen M (2005). Production of red pigments by the insect pathogenic fungus *Cordyceps unilateralis *BCC 1869. J Ind Microbiol Biotechnol.

[B31] Cho YJ, Park JP, Hwang HJ, Kim SW, Choi JW, Yun JW (2002). Production of red pigment by submerged culture of *Paecilomyces sinclairii*. Lett Appl Microbiol.

[B32] de Hoog GS, Guarro J, Gene J, Figueras MJ (2000). General techniques. Atlas of clinical fungi.

[B33] Mapari SAS, Hansen ME, Meyer AS, Thrane U (2008). Computerized screening for novel producers of *Monascus*-like food pigments in *Penicillium *species. J Agric Food Chem.

[B34] Mapari SAS, Meyer AS, Thrane U (2008). Evaluation of *Epicoccum nigrum *for growth morphology and production of natural colorants in liquid media and on a solid rice medium. Biotechnol Lett.

[B35] Hajjaj H, Klaebe A, Loret MO, Goma G, Blanc PJ, Francois J (1999). Biosynthetic pathway of citrinin in the filamentous fungus *Monascus ruber *as revealed by ^13^C nuclear magnetic resonance. Appl Environ Microbiol.

[B36] Frisvad JC, Andersen B, Thrane U (2008). The use of secondary metabolite profiling in chemotaxonomy of filamentous fungi. Mycol Res.

[B37] Nielsen KF, Smedsgaard J (2003). Fungal metabolite screening: database of 474 mycotoxins and fungal metabolites for dereplication by standardized liquid chromatography-UV-mass spectrometry methodology. J Chromatogr A.

[B38] Samson RA, Seifert KA, Kuijpers AF, Houbraken JA, Frisvad JC (2004). Phylogenetic analysis of *Penicillium *subgenus *Penicillium *using partial β-tubulin sequences. Stud Mycol.

[B39] Raper KB, Thom C (1949). Manual of the Penicillia.

[B40] Pitt JI (1979). The Genus Penicillium and its Teleomorphic States, Eupenicillium and Talaromyces.

[B41] LoBuglio KF, Taylor JW, Reynolds DR, Taylor JW (1993). Molecular phylogeny of *Talaromyces *and *Penicillium *species in subgenus *Biverticillium*. The fungal holomorph: mitotic, meiotic and pleomorphic speciation in fungal systematic.

[B42] Lin TF, Yakushijin K, Büchi GH, Demain AL (1992). Formation of water-soluble *Monascus *red pigments by biological and semi-synthetic processes. J Ind Microbiol.

[B43] Blanc PJ, Loret MO, Santerre AL, Pareilleux A, Prome D, Prome JC, Laussac JC, Goma G (1994). Pigments of *Monascus*. J Food Sci.

[B44] Hajjaj H, Klaebe A, Loret MO, Tzedakis T, Goma G, Blanc PJ (1997). Production and identification of N-glucosylmonascorubramine from *Monascus ruber *and occurrence of electron donor-acceptor complexes in these pigments. Appl Environ Microbiol.

[B45] Jung H, Kim C, Shin CS (2005). Enhanced photostability of *Monascus *pigments derived with various amino acids via fermentation. J Agric Food Chem.

[B46] Frisvad JC, Thrane U, Samson RA, Hoekstra ES, Frisvad JC (2004). Mycological media for food- and indoor fungi. Introduction to food- and airborne fungi.

[B47] Smedsgaard J (1997). Micro-scale extraction procedure for standardized screening of fungal metabolite production in cultures. J Chromatogr A.

[B48] Nielsen KF, Gräfenhan T, Zafari D, Thrane U (2005). Trichothecene production by *Trichoderma brevicompactum*. J Agric Food Chem.

